# Supplementary material for: Are carbon emissions trading and green financial instruments synergistic? -Comprehensive quantitative research based on content analysis
Source: PLoS One. 2024 Mar 7;19(3):e0298601. doi: 10.1371/journal.pone.0298601 (PMC10919601; doi:10.1371/journal.pone.0298601)
Supplement: S1 Data — (ZIP) [file pone.0298601.s001.zip › Data/地级市Z_F工作报告.zip!/║■─╧/╥μ╤⌠╩╨/└┤╫╘╣1⁄2╓┌║┼í╛┬φ┐╦╩2╛▌═°í┐_2015.docx]

各位代表：

　　现在，我代表市人民政府向大会作政府工作报告，请予审议，并请市政协委员和列席会议的同志提出意见。

　　一、2014年政府工作

　　2014年，在省委、省政府的坚强领导下，按照中共益阳市委的决策部署，全市人民团结拼搏，务实奋进，实现了经济的平稳较快发展和社会大局的和谐稳定。全年完成生产总值1253.2亿元（预计数，下同），比上年增长10.8%；完成公共财政总收入94.9亿元，增长10.3%；完成固定资产投资1030.6亿元，增长22.3%；实现社会消费品零售总额454.2亿元，增长12.8%；城乡居民人均可支配收入15260元，增长10.4%，其中城镇居民人均可支配收入20688元，增长9.3%，农村居民人均可支配收入11304元，增长11.6%。

　　（一）产业结构调整优化。三次产业结构比由上年的19.0:45.2:35.8调整为18.7:44.1:37.2。农业生产稳步发展。全市实现农林牧渔业增加值226亿元，比上年增长4.3%。粮食生产面积稳定增长，蔬菜、黑茶、生猪、水产等产业健康发展。工业经济平稳增长。完成规模工业增加值537.5亿元，增长11.6%。新进规模工业企业107家，全市规模工业企业达到922家。新增高新技术企业13家，完成高新技术产品增加值146亿元，增长25%。新增湖南名牌14个。第三产业逐步提升。实现第三产业增加值465.8亿元，增长13.5%。交通运输、现代金融、休闲旅游、电子商务、信息咨询、现代物流等服务业较快发展。

　　（二）项目建设扎实推进。26个省级重点项目、市“双百工程”的“百大项目”均超额完成年度投资计划。交通建设大会战强力推进。完成交通基础设施建设投资90.08亿元。其中高速公路完成投资37.87亿元，增长55.7%。319国道益阳南线高速等项目正在加快建设，益娄高速、益马高速均已实质性开工，益南高速、马安高速即将实质性开工，安官高速、宁韶高速、益平高速、益长高速复线等项目前期工作进展顺利。石长铁路复线益阳段正在加快推进。益阳大桥开工建设。资阳大桥、黄茅洲大桥、S317茅东公路、S317资北干线建成通车。园区建设大会战取得实效。全市省级以上工业园区完成基础设施建设投资122亿元，新开工工业项目104个，新投产工业项目102个，新建标准化厂房122.3万平方米；完成规模工业增加值295亿元，比上年增长13.6%，占全市规模工业增加值的比重达56.2%。龙岭工业园发展经验在全省推介，并获批为省级“飞地经济”试点园区。

　　（三）改革开放不断深化。各项改革逐步推进。全市9大类55项改革已经完成15项，其他改革事项正在抓紧推进。行政审批制度改革继续深化，市本级行政许可和管理服务项目减少137项，精简50.5%，保留项目为全省最少。政府机构改革有序推进。注册资本登记制度改革效果明显，市场主体达到19.4万家，比上年增长20.9%。农村土地承包经营权确权颁证率先试点。农村金融改革探索取得新成效。市直国有企业改制任务基本完成。财税、教育、司法、文化、医疗卫生等各项改革不断深化。引资争资取得成效。引进市域外资金392亿元，形成固定资产投资322.2亿元，比上年增长19%。其中，实际利用外资2.08亿美元，增长22.7%。争取各类资金170.2亿元，增长10%。对外贸易与交流合作得到加强。完成进出口总额35.7亿元，比上年增长18.7%，其中出口32.5亿元，增长18.6%。

　　（四）发展方式逐步转变。生态建设扎实推进。开展国家生态市创建工作，安化县、南县、沅江市纳入国家和省级重点生态功能区。加大了城市规划区自然山体水体保护力度。大力发展高效生态林业，完成各类造林24万亩。环境保护得到加强。重点推进重金属污染防治、大气污染防治和“两河”治理，中心城区空气质量优良率达93.7%，洞庭湖和资江益阳段10个断面全部达到国家地表水Ⅲ类标准，主要饮用水源监测合格率达到100%。节能减排效果明显。单位生产总值和规模工业增加值能耗分别下降5.5%和10.6%，超额完成省定目标。主要污染物减排指标完成省定任务，其中二氧化硫减排提前完成“十二五”减排目标。“两型”示范创建工作全面推进。“两型”社会示范区共完成各类投资80多亿元。东部新区鱼形山大道和内环线建设进展顺利，江南古城项目第一期开街。科技创新能力不断增强。实现专利授权980件。我市获批为信息惠民国家试点市。国家黑茶产品质量监督检验中心获准筹建。中国黑茶产业技术联盟创新服务平台、沅江船舶制造产业集群公共服务平台成为湖南省首批科技创新与服务平台。

　　（五）城乡统筹深入推进。城乡统筹工作力度加大。县域统筹城乡专项规划全面完成，48个乡镇完成村镇布局规划。土地流转面积301.7万亩，其中耕地流转面积197.7万亩。“四位一体”村级基层组织治理机制覆盖99.7%的行政村。续建和新建农民集中居住区36个。农村清洁工程、涉农资金整合等工作深入推进。城镇建设步伐加快。全市完成城镇基础设施建设投资81.04亿元，比上年增长21.7%。完成了《益阳市城市总体规划（2014－2020）》修改并报省政府批准同意。中心城区完成基础设施建设投资43.2亿元，垃圾焚烧发电等“两供两治”和“两房两棚”项目建设扎实推进。全市共完成保障性住房5.6万余套，资阳区棚改模式在全省推广。创卫成果进一步巩固，交通管理模范城市创建扎实有效。县城建设加快推进，桃江县城西环线、沅江“五湖连通”二期工程、南县“四纵四横”道路改造、安化南区建设等项目进展顺利。成功申报国家级重点镇12个。农村基础设施建设得到加强。完成农村公路建设575公里。完成474个村的农网改造升级。完成水利建设投资21.03亿元。

　　（六）民生民利持续改善。省政府下达的15项实事26个指标全面完成。就业形势保持稳定。新增城镇就业3.7万多人，新增农村劳动力转移就业2.4万多人，新增创业4万多人，带动就业10.3万多人。社会保障体系不断完善。五大社会保险共征收保险费29.5亿元，发放68亿元，各项社保待遇按时足额发放。基层人社平台建设全面完成。城乡居民医疗保险和养老保险实现统筹，城乡居民大病保险试点正式启动。1.1万名被征地农民纳入社保。城乡低保和农村五保标准进一步提高。惠民政策全面落实。通过“一卡通”直接发放到农户的各类惠农补贴13.5亿元，取消、降标或免征涉企涉农收费项目减轻群众负担3000多万元。落实城镇低保与特困户价费优惠2000多万元。片区扶贫开发成效明显，减少贫困人口5.63万人。完成移民避险搬迁安置2997人。社会事业全面发展。加大教育投入，建成84所合格学校和18所农村公办幼儿园，改善了174所农村学校和12所职业学校办学条件。中心城区新增学位3460个。教育招生改革取得突破。校车安全管理得到加强。公共文化服务体系不断健全，益阳“文博现象”的影响力继续提升，兔子山遗址获评年度“全国十大考古新发现”，历史文化名城申报工作稳步推进，文化惠民活动扎实开展。公共卫生服务水平逐步提高，重大疾病防控协调推进，医疗质量与安全管理不断加强。食品药品安全监管和产品质量监管力度加大。人口计生工作得到加强，低生育水平保持稳定。群众体育和竞技体育取得新成绩。国土资源管理工作取得成效，重大项目用地需求基本得到保障，连续15年实现耕地占补平衡。新闻媒体的舆论导向和宣传影响不断增强。残疾人事业得到长足发展。国防动员组织指挥能力不断提高，人民防空建设扎实推进，优抚安置工作落到实处。统计、审计、档案、保密、对台、科协、地方志、红十字会、消防、民族宗教、工商、供销、烟草、地震、气象、水文、通信、住房公积金、公共资源管理、无线电管理、老龄等工作取得新进步。工会、共青团、妇联等群团组织及各民主党派、工商联、无党派人士在经济社会发展中发挥了积极作用。

　　（七）社会大局和谐稳定。精神文明建设深入推进。社会主义核心价值观宣传教育和文明城市创建、基层文明创建活动扎实开展。评选表彰了第三届全市道德模范。志愿服务（义工）工作取得实效。民主法制建设不断加强。顺利完成了第九次村民委员会换届选举。社区治理和服务机制逐步完善。法治益阳建设加快推进，普法依法治理工作取得成效。安全生产形势持续稳定好转。高度重视安全生产工作，没有发生重大及以上安全生产事故。三大高危行业共关闭退出落后小企业49家。安全生产工作获得省先进单位称号。综治维稳工作成效明显。平安益阳建设扎实推进，社会治安形势保持稳定，成为全省年度考核评估先进市。落实维稳工作第一责任，创新群众工作机制，扎实抓好矛盾纠纷排查调处，保持了社会大局的持续稳定。

　　（八）自身建设得到加强。政府及其部门扎实开展党的群众路线教育实践活动，深入推进“三访三化”专项活动，干部作风进一步好转。主动接受人大及其常委会的法律监督、工作监督，自觉接受人民政协的民主监督，办理市人大代表建议146件、政协委员提案76件，代表、委员满意率稳步提高。抓好市长热线、信访接待等工作，解决了一批事关人民群众切身利益的实际问题。全面推进政务公开和网上政务服务，行政效能稳步提升。认真履行“一岗双责”，加强执纪问责，开展电视问政、网络问政和述廉评议，严查腐败案件，政风行风进一步好转。

　　各位代表，过去一年的成绩，是省委、省政府和中共益阳市委正确领导的结果，是全市人民团结拼搏的结果，是社会各界关心支持的结果。在此，我代表市人民政府，向全市人民，向中央、省属驻益单位，向驻益人民解放军和武警指战员，向所有关心、支持益阳改革发展的各界朋友，表示衷心的感谢！

　　在肯定成绩的同时，我们也清醒地认识到当前我市经济社会发展面临的困难和问题。主要是：经济总量不大，产业龙头不强，产业结构不优；部分经济指标增速没有达到年初预期；财政收支矛盾突出；基础设施仍然薄弱；保障和改善民生任务艰巨；房地产、政府性债务、金融等领域的风险可能累积叠加；在改革开放、政府职能转变、作风建设及环境优化方面需要加大力度，等等。对这些问题，我们将在今后的工作中认真加以解决。

　　二、2015年主要工作

　　2015年是全面推进依法治国的开局之年，是全面深化改革的关键之年，是全面完成“十二五”规划的收官之年，做好政府各项工作意义重大。

　　2015年政府工作的总体要求是：全面贯彻党的十八大和十八届三中、四中全会精神，坚持全面建成小康社会总目标，坚持稳中求进工作总基调，主动适应发展新常态，抢抓洞庭湖区域发展新机遇，继续强化干事、修路、招商、创业“四大举措”，深入推进工业立市、农业稳市、三产兴市、生态美市、人才强市“五大战略”，促进经济持续健康发展和社会和谐稳定。

　　2015年全市经济社会发展的预期目标是：生产总值增长10%左右，一般公共预算收入增长10%左右，城乡居民人均可支配收入增长10%左右，固定资产投资增长20%以上，社会消费品零售总额增长12%以上，规模工业增加值增长11%以上，单位生产总值能耗、主要污染物排放量、人口自然增长率控制在省定指标以内。

　　重点抓好九个方面的工作：

　　（一）强力推进洞庭湖生态经济区建设，不断提升发展水平

　　加快洞庭湖生态经济区建设。加强规划衔接。紧紧围绕解决“粮”、“水”、“路”等关键问题，突出生态环保、饮水安全、综合交通体系、特色产业发展、特色城镇建设五大工作重点，把洞庭湖生态经济区建设规划和项目及时落实到“十三五”规划及全面小康建设规划之中。突出项目实施。务实推进省“511工程”项目库中益阳区域项目建设；千方百计落实我市已经规划的566个重大项目；扎实推进全面小康22个重点项目建设；层层落实“双百工程”重大项目建设。继续认真抓好桃花江核电项目的前期工作。抓好项目协调。加大对重大项目的统筹协调力度，明确责任分工，按照前期项目、新开工项目、在建项目分类分步推进，形成开工一批、竣工一批、储备一批的良性循环。

　　全面推进生态建设。深入推动国家生态市创建工作。严格落实《益阳市城市规划区自然山体水体保护与利用管理办法》。加强湿地保护，做好南洞庭湖晋升国家级自然保护区申报工作。大力推进“矿山复绿”行动。全面推进高效生态林业建设，加强薄弱地区的生态恢复，保护生物多样性。完成造林23万亩，其中重点工程造林10.6万亩。抓好“两型”示范创建，进一步加快东部新区建设。

　　加强节能减排和环境保护。认真贯彻新《环保法》，强化环保工作责任。着力调整产业结构，淘汰落后产能。突出抓好减排项目落实，实施排污权的储备、有偿使用和交易，实现主要污染物排放量持续下降。深入推进“两河”治理，抓紧谋划实施中心城区“两年治污计划”。继续加强农村面源污染治理、城镇污水垃圾处理，抓好重金属污染、大气污染和水污染防治。

　　（二）强力推进改革开放，全面激发发展活力

　　深化重点领域改革。继续抓好已经启动的各项改革的成果巩固和纵深推进。深化行政审批制度改革。认真做好国务院和省政府下放行政审批事项的承接落实，继续抓好行政审批事项的取消和下放。抓好政府机构改革。进一步精简政府机构，规范机构设置，完善市县政府功能，理顺条块关系。深化财税体制改革。认真贯彻新《预算法》，推进预算管理改革，健全全口径预算管理体系；推进财政绩效管理改革；推进“营改增”改革；继续加强税收协控联管。深化投融资体制改革。积极研究和采用PPP模式，拓宽政府投融资路子，激活民间资本参与基础设施和公用事业建设。抓好农村综合配套改革。加大农村土地确权颁证力度，推进农村产权制度改革；进一步完善实施方案，抓好现代农业综合配套改革试验。推进政府购买服务。在保障性住房后续管理、公路养护、法律援助、投资评审等服务项目购买上取得突破。巩固国有企业改革成果。完成公车改革工作任务。

　　引导招商引资创新发展。力争全市引进市域外资金形成固定资产投资360亿元，实际利用外资2.3亿美元，实现新引进、新开工和新投产项目增长20%以上。突出产业招商，瞄准世界和国内500强、央企、行业龙头企业等战略投资者，加快战略性项目的引进，同时注重引进中小配套企业；强化园区招商，完善各园区产业定位，引导各园区错位发展，科学引进重点工业项目和高端三产业项目；抓好集群招商，大力引进行业地位高、竞争力强的核心企业进驻，完善产业链，形成产业集聚效应。充分利用专业招商、商会招商、协会招商、以商招商、乡情招商等手段，提升招商引资实效。

　　积极开展对外贸易与交流合作。加快益阳口岸机构建设步伐，提高贸易便利化程度。推进出口基地和外贸公共平台建设，引导出口企业做精做细传统市场，做大新兴市场。大力发展加工贸易。加快发展跨境电子商务、服务贸易等新兴贸易方式，促进网上贸易倍增。加强与国际友好城市的实质性交流与合作。

　　（三）强力推进产业发展，继续做大经济总量

　　全力推进新型工业化。做大工业经济总量。坚持把新型工业化作为加快益阳发展的重中之重，确保实现规模工业增加值585亿元。努力提升工业发展质量。围绕先进装备制造、食品加工、电子信息等优势产业，大力发展中小企业，培育发展一批主营业务收入上10亿元、50亿元的龙头骨干企业，推动形成产业集群；加强纺织、冶金、建材、竹木加工等传统产业提质改造；扶持发展生物医药、新能源、新材料等战略性新兴产业。积极推进企业上市。力争1家以上企业在主板挂牌交易，推动2家以上企业挂牌“新三板”，着力引进产业投资基金、私募股权基金等风险资本参与孵化拟上市企业。大力推进自主创新。实现专利申请量突破2000件，高新技术产品增加值增长20%以上，全社会研发投入增长幅度高于全省平均水平，让更多的研发成果及时转化为现实生产力。完善以企业为主体的技术创新体系，引导企业加强品牌建设，提升核心竞争力。

　　大力发展现代农业。继续抓好粮食生产。稳定粮食生产面积620万亩以上，粮食总产240万吨以上。突出抓好特色产业。做大做强蔬菜产业；继续做强做优黑茶产业，办好第三届黑茶文化节；抓好水产养殖、加工和出口，进一步提高发展质效；大力推进花卉苗木产业转型升级；抓好笋竹、油料、中药材等产业发展，支持芦笋产业做大做强和打造特色品牌。着力提升农业产业化水平。积极推进农村土地流转和适度规模经营。大力发展农产品加工业，推进一产业和二、三产业融合发展。培育年销售收入过5亿元的龙头加工企业7家、过10亿元的3家。积极培育新型农业经营主体。进一步发展种养大户、家庭农场、农民专业合作社和农业企业，鼓励和引导社会资本投资现代农业，新创建县级以上示范合作社80家、示范家庭农场100家。提高农产品质量安全水平。加强农产品质量安全监管体系建设，努力实现农产品质量安全监管乡镇、村组全覆盖，确保不发生重大农产品质量安全事故。强化农业科技支撑。加强农业科技研究与推广应用，健全农业社会化服务体系，提高农机化水平。

　　加快发展现代服务业。大力发展电子商务产业。认真落实支持电子商务发展政策，突出抓好益阳高新区中南电商园等平台建设，实现新增规模以上电子商务企业50家以上，电子商务交易额达到60亿元以上。加快发展生产性服务业。继续大力发展现代金融。加强金融机构建设，继续引进商业银行，支持金融机构创新信贷产品，进一步提高信贷投放能力；抓好省农村金融改革试点市建设；加强金融生态环境建设，切实防范金融风险；促进保险行业规范发展。加快现代物流业发展。着力完善交通运输体系，加强物流园区和专业市场建设。进一步发展服务外包产业。促进消费性服务业发展。促进房地产业健康发展。积极发展文化旅游业，推进旅游大项目、精品项目开发建设和旅游基础设施完善，提升旅游产品竞争力。加快健康服务、养老服务、家政服务等新型消费服务业发展。改造提升传统服务业。加快对餐饮、住宿等传统服务业的改造升级，提高传统服务业质量水平。

　　（四）强力推进“两大会战”，不断夯实发展基础

　　扎实推进交通建设大会战。全年完成交通建设投资130亿元以上。加快在建项目建设。确保牛田至朱良桥公路（一期）、宁家铺至泉交河公路建成投入使用；力争G319益阳南线高速、常安和安邵高速益阳段建成通车；完成益娄高速益阳段、益马高速、S225安化段、S217河南线沅江段和大通湖段、S228沅菁线沅江段、梅城至黄柏界公路、益阳大道西延线、益阳大桥、安化大桥等在建项目年度投资任务。进一步加快危桥改造、渡口改造、渡改桥等项目的建设步伐。推动一批项目开工建设。确保益南高速、马安高速2个项目实质性开工，力争益长高速复线、安官高速、宁韶高速益阳段3个项目开工，争取S230杨林坳至高桥公路等7个国省干道项目动工建设。抓好项目前期工作。努力推动益娄铁路扩能改造、常岳九铁路等项目纳入国家层面规划实施；力争常益长高铁、安张衡铁路、华益铁路纳入国家铁路总公司“十三五”发展规划；积极推进中心城区通用机场、安新高速、益平高速、S228沅菁线资阳段等项目的前期工作。

　　大力推进园区建设大会战。加强园区项目建设。认真对接省“135工程”，力争园区新建标准化厂房150万平方米，完成基础设施投资100亿元，实现园区新签约项目140个以上，新开工项目100个以上，新投产项目100个以上。提升园区运行质量。坚持以发展质量和效益为导向，创新园区发展思路，实现园区规模工业增加值增长13.6%以上，占全市规模工业增加值的比重达到57.4%以上，工业税收增长12%以上，土地产出率达到700万元/亩。突出园区发展特色。瞄准电子信息、汽车零部件、船舶制造、生物医药、黑茶生产等产业发展，着力打造10个特色创业园区，引进和培育创新企业300家。增强园区承载能力。加大园区生产要素的保障和供给，加强园区孵化器、创新创业平台和配套设施建设，改善园区生产生活环境。

　　（五）强力推进新型城镇化，努力促进城乡统筹

　　统筹城乡发展目标。坚持“以人为本、产城融合”的发展理念，突出中心城区和县城发展的引领作用，努力提高建管水平。全市城镇化水平达到46%。完成城镇基础设施建设投资95亿元，其中中心城区完成投资43.8亿元。县城扩容提质和重点镇建设取得新的突破。每个区县（市）高标准建设一个新农村示范村，完成190个行政村村庄人居环境整治和13个美丽乡村示范点建设。

　　统筹城乡规划。坚持“多规融合”原则，完成市县城市总规修改修编，做好控规调整和审批工作，抓好排水防涝、城市综合体等专项规划编制，全面完成镇（乡）域村镇布局规划，全面启动村庄规划编制，努力实现城乡规划全覆盖。全面建立乡镇规划例会制度。

　　统筹城乡建设。加快推进城镇建设。推进城市排污治理、地下管廊、绿化亮化等重点建设项目。加强“两供两治”建设，切实保障城镇供水质量，加快实施“县县通”天然气工程，强力推进龙岭污水处理厂等9个污水处理项目建设，完成市垃圾焚烧发电项目建设，县以上城镇生活污水处理率、垃圾无害化处理率分别达91%、92%，供水普及率、燃气普及率分别达96%、98%。加大中心城区“城中村”改造力度，加快推进马良村、毛家塘村、七里桥村等的联片改造。实现省级园林城市创建目标，开工建设资阳公园、云雾山体育主题公园，启动将会龙公园与秀峰公园捆绑打造4A级景区工作。强化农村基础设施建设。完成水利建设投资22.58亿元，继续推进水利十大工程建设。加快农村饮水安全工程建设，全面完成“十二五”农村饮水安全任务。加大农村土地综合整治力度，严守耕地红线和林地生态红线。加强农村公路和水运基础设施的建设与管理养护。推进智能电网建设，继续抓好农网升级改造。

　　统筹城乡管理。协调推进国家交通管理模范城市、国家环保模范城市、国家历史文化名城的创建、申报工作，不断提升城市品位。强化城市综合执法，加大查违拆违力度。进一步理顺城管执法体制，稳步推进“城管进社区”、“城管进乡镇”，推动城市管理重心下移。扎实推进数字城管系统二期建设。稳步推进城区停车泊位收费管理工作。积极支持重点镇创建国家（省）级文明卫生城镇，每个区县（市）均完成1个以上创建任务。抓好农民建房管理。深入推进农村清洁工程和环境整治工程。

　　统筹城乡公共服务。抓好城乡供水、供电、供气、通信等基础设施的对接整合，大力推进信息惠民十大工程，加快文化、教育、卫生、体育、就业、社保等公共服务设施的均等化建设，提升城乡公共服务一体化水平。

　　（六）强力推进民生改善，提升市民幸福指数

　　推动就业创业。坚持以创业带就业，实现新增创业主体1.2万户，城镇新增就业3.4万人，新增农村劳动力转移就业1.7万人，城镇登记失业率控制在4.5%以内。重点解决高校毕业生就业和结构性失业问题。巩固基层人社平台建设成果，做好人力资源信息监测、失业动态监测等基础工作。健全面向全体劳动者的职业培训制度。

　　做好社会保障工作。抓好扩面征缴，全年实现各项社会保险新增参保3.5万人，征缴各项社会保险费33亿元，按时足额落实各项社保待遇。完成100万张社保卡发放任务。全面实施城乡居民大病保险。落实园区养老保险缴费费率过渡试点政策，启动实施机关事业单位养老保险制度改革工作。

　　完善社会救助服务体系。加强基层社会救助能力建设，城乡低保保障标准分别由每月330元、165元提高到350元、175元，农村五保户分散供养标准由每年2640元提高到2800元。加快推进社会养老服务体系建设，全面落实老年人优待政策。推动慈善事业长足发展。

　　强化保障性安居工程建设。积极筹集资金，创新建设模式，加快推进以公租房、城市棚改、国有工矿棚改为重点的保障性住房建设，完成保障性住房3.98万套（户）。继续完善相关配套政策，强化住房分配管理，规范后续管理，让老百姓住得安心。“整镇整村”推进农村危房改造，完成渔民上岸安居任务。

　　抓好扶贫和移民工作。加大武陵山片区扶贫攻坚力度，落实精准扶贫措施，全年减贫5万人以上。继续加大对相对落后地区全面小康建设的支持。加快移民集中区基础设施建设和产业发展。

　　（七）强力推进社会事业发展，促进社会全面进步

　　办好人民满意的教育。大力推进教育强市建设，促进各级各类教育均衡发展。高质量全面完成义务教育合格学校建设。完善中心城区学校规划和建设机制，加快农村薄弱学校改造。大力支持社会力量办学。全面实施素质教育，推进课堂教学改革，加强学校德育工作。深化学生综合评价改革，建立全面、科学的教育质量监控体系。进一步完善招生改革，继续实行阳光招生。加快推进职业教育改革，引导普职分流。推动益阳高等教育更好地服务地方经济社会发展。深化教师人事制度改革，加强农村教师和专业教师队伍建设。

　　完善医疗保障体系。全面推进县级公立医院综合改革，完成14家县级公立医院改革任务。运用PPP模式启动中心城区3家公立医院股份制改造。鼓励和引导社会资本举办医疗机构。完善重特大疾病救助机制。完成省级区域卫生信息平台试点市建设。逐步实施分级诊疗制度，加强县、乡、村三级医疗和公共卫生服务能力建设，提高全民医疗保障水平。

　　积极发展文化事业。加强公益性文化基础设施建设，启动“五馆三中心”建设；加快兔子山遗址、羊舞岭遗址公园、石码头历史文化街区规划建设和项目申报，完成明清古巷抢救性修缮，完成万里茶路申报世界文化遗产的益阳段调勘。进一步推进文化惠民，继续实施花鼓戏振兴工程。

　　加强精神文明建设。认真开展培育和践行社会主义核心价值观工作，深入推进群众性精神文明创建活动，开展第四届道德模范评选，提高全民思想道德素质。扎实抓好全国文明城市创建工作，争取通过三年努力实现创建目标。

　　统筹抓好其他各项工作。继续大力实施“十百千万”人才工程，完善人才引进、培养、使用的科学机制；认真实施优秀人才支持计划，加大对创新创业人才的扶持力度；支持各民主党派、工商联、无党派人士深入实施“同心工程”。抓好人口和计划生育“三网融合”工作，着力夯实基层基础，稳定低生育水平，并以此为平台创新基层社会管理。深入开展全民健身运动。切实加强食品药品安全监管。强化质量建设和质量安全监管。重视抓好气象、水文基础项目建设，提升防灾减灾能力。积极推进非公经济组织住房公积金制度建设。切实加强残疾人“两个体系”建设，大力实施“贫困残疾人救助工程”。支持工会、共青团、妇联等群团组织依照章程开展工作，全面实施好妇女儿童发展规划，积极推动志愿者（义工）事业发展。加强全民国防教育，抓好国防动员和后备力量建设，支持武警、消防部队建设。切实抓好国家安全、统计、工商、质监、供销、烟草、民族宗教、外事侨务、对台、人防、档案、保密、地方志、老龄、红十字会、关心下一代等各项工作。

　　（八）强力推进社会治理，维护社会和谐稳定

　　加强民主法制建设。继续加强“四位一体”村级组织建设，进一步完善村务公开，全面开展农村社区建设试点。深入开展和谐社区创建活动。坚持法制宣传教育与法治实践相结合，争创全国法治先进城市。加快推进公共法律服务体系建设，扩大法律援助范围，提升法律援助服务质量。抓好区县（市）社区矫正中心建设试点，提高社区矫正工作水平。

　　营造和谐稳定的社会环境。坚持用法治思维统揽维稳工作，不断巩固和扩大“三访三化”专项活动成果，加强人民调解工作，深入排查化解矛盾纠纷，抓好社会稳定风险评估，进一步增强舆情应对和应急处突能力。加强劳动监察执法和劳动人事争议调解仲裁工作，全面落实农民工工资保障金制度，构建和谐劳动关系。加强社会治安综合治理，强化“天网工程”、“巡防工程”建设，完善社会治安体系，严厉打击各类违法犯罪活动，增强人民群众安全感。

　　认真抓好安全生产工作。认真贯彻新《安全生产法》，强化安全生产红线意识，实施最严格的安全生产责任制度。夯实安全生产基层基础工作，加强安全生产监管能力建设。进一步强化安全生产源头治理，继续深化高危和落后小企业关闭整顿工作。继续推进安全生产隐患的排查治理，提升企业自身安全保障水平。预防和减少安全生产责任事故，有效遏制较大事故，坚决杜绝重大及以上安全生产事故发生。

　　（九）强力推进依法行政，加强政府自身建设

　　进一步加强法治政府建设。自觉接受人大及其常委会的法律监督、工作监督和政协的民主监督，推进协商民主，认真办理人大代表建议和政协委员提案，主动接受舆论监督和社会监督。依法全面履行政府职能，规范行政权力运行。建立政府权力清单、负面清单、责任清单和政府服务网等“三单一网”，充分发挥市场对资源配置的决定性作用。建立健全科学民主决策机制，重大行政决策严格遵循公众参与、专家论证、风险评估、合法性审查、集体讨论决定等法定程序。加强政府制度建设，提高规范性文件质量。加强行政执法能力建设，强力推进综合执法。加强行政复议规范化建设，进一步提高行政复议办案能力。加快建设专业化、高水平的政府法制队伍。

　　进一步加强民本政府建设。继续抓好为民办实事工作，全面完成省政府下达的为民办实事的各项工作任务。充分发挥市长热线、信访接待等民生沟通主渠道作用，认真解决群众关注的难点、热点问题。主动加强重大民生问题调研，完善保障和改善民生的发展规划，有计划有步骤地持续推进民生问题解决，为人民群众提供更多更好的公共产品和公共服务，让人民群众得到更多实惠，生活得更加幸福、更有尊严。

　　进一步加强效能政府建设。努力提高政务服务效率。进一步改进文风、会风，政府系统下发的规范性文件、召开的全市性会议数量继续减少30%以上。最大限度减少各类检查、评比和达标活动。大力推进政务公开与网上政务服务，加大政府信息主动公开力度，加强政务中心和公共资源交易平台建设，加快电子政务建设，不断简化审批流程，扎实推进中心城区建设项目并联审批和联合验收。全面加强行政效能监察，整治低效作为、办事拖拉等问题。切实改善企业发展环境。严格依法依规开展涉企执法和检查，坚决杜绝执法扰企行为，最大限度为企业松绑减负，让企业引得进、落得下、长得大。大力整治项目建设环境。认真落实重点项目建设工作责任制，严厉打击非法阻工、强揽工程、强装强卸等违法行为。

　　进一步加强廉洁政府建设。强化政府及部门班子成员党风廉政建设主体责任和“一岗双责”，认真落实各级关于加强党风廉政建设的有关规定。深入推进财政预算公开，严控“三公”经费支出，降低行政成本。加大审计和审计结果公告力度。加强监督检查，深化专项治理，切实纠正行业不正之风。深入推进反腐倡廉，坚决查处腐败案件，努力营造良好政风。

　　科学编制“十三五”规划是今年的一项重大任务。要从我市实际出发，深入研究经济新常态的发展规律，准确把握面临的重大机遇和挑战，科学提出今后五年的发展目标和任务，使规划成为凝聚力量、指引发展的任务书、路线图。

　　各位代表，益阳正处在加快发展、后发赶超的关键时期，时代赋予了我们新的历史重任。让我们在中共益阳市委的坚强领导下，全面解放思想，合力攻坚克难，真抓实干，开拓进取，为加快后发赶超、建设丽都益阳、全面建成小康社会而努力奋斗！
